# Calcitonin gene related peptide monoclonal antibody treats headache in patients with active idiopathic intracranial hypertension

**DOI:** 10.1186/s10194-020-01182-7

**Published:** 2020-09-25

**Authors:** Andreas Yiangou, James L. Mitchell, Vivek Vijay, Olivia Grech, Edward Bilton, Gareth G. Lavery, Claire Fisher, Julie Edwards, Susan P. Mollan, Alexandra J. Sinclair

**Affiliations:** 1 Metabolic Neurology, Institute of Metabolism and Systems Research, College of Medical and Dental Sciences, University of Birmingham, Birmingham, B15 2TT UK; 2 Centre for Endocrinology, Diabetes and Metabolism, Birmingham Health Partners, Birmingham, B15 2TH UK; 3 Department of Neurology, University Hospitals Birmingham NHS Foundation Trust, Birmingham, B15 2TH UK; 4 Birmingham Neuro-Ophthalmology, Ophthalmology Department, University Hospitals Birmingham NHS Foundation Trust, Birmingham, B15 2TH UK

**Keywords:** CGRP monoclonal antibody, Headache, Idiopathic intracranial hypertension, Papilloedema, Raised intracranial pressure

## Abstract

**Background:**

Headache is the dominant factor for quality of life related disability in idiopathic intracranial hypertension (IIH) and typically has migraine-like characteristics. There are currently no evidence-based therapeutics for headache in IIH, and consequently this is an important unmet clinical need.

**Case series:**

We report a series of seven patients in whom headaches were the presenting feature of IIH and the headaches had migraine-like characteristics, as is typical in many IIH patients. Papilloedema settled (ocular remission) but headaches continued. These headaches responded markedly to erenumab, a monoclonal antibody targeted against the calcitonin gene related peptide (CGRP) receptor. Of note, there was a recurrence of raised ICP, as evidenced by a return of the papilloedema, however the headaches did not recur whilst treated with erenumab.

**Conclusions:**

Those with prior IIH who have their headaches successfully treated with CGRP therapy, should remain under close ocular surveillance (particularly when weight gain is evident) as papilloedema can re-occur in the absence of headache. These cases may suggest that CGRP could be a mechanistic driver for headache in patients with active IIH.

## Introduction

Idiopathic intracranial hypertension (IIH) is a chronic debilitating disease characterised by raised intracranial pressure (ICP) that typically occurs in young, obese women [1]. There is evidence of rapidly increasing incidence (350% increase in 10 years), in line with global obesity trends [1, 2]. Disability in IIH is predominantly driven by debilitating headache [3]. Headaches in IIH most frequently have migraine-like characteristics [4]. Therapeutic strategies to prevent headache in IIH are an unmet need [1, 4, 5].

We report seven patients who presented with headaches in the setting of raised ICP and who met the diagnostic criteria for IIH [6], whose IIH remitted and headaches persisted. Following trials of conventional headache therapies that failed to control their headache symptoms over 12 months, they were subsequently treated with a monoclonal antibody targeted against calcitonin gene related peptide (CGRP) receptor, erenumab (Aimovig®, Novartis). We discuss the possible pathophysiological implications, and importantly the clinical considerations. These cases may suggest a role of CGRP in headache attributed to IIH. Importantly these cases suggest that when headaches attributed to IIH are controlled with medical therapy, ocular examinations should continue to ensure papilloedema (optic disc swelling secondary to raised ICP) does not recur silently.

## Findings

Following IIH diagnosis all patients were treated according to the IIH consensus guidelines with weight management advice and acetazolamide therapy where appropriate [1, 7]. The mean (SD) duration since the IIH diagnosis was 2.4 years (1.5). All cases presented with increased headaches at the time of initially elevated ICP and IIH diagnosis. Their headaches persisted, even after resolution of papilloedema, and had migraine-like characteristics (photophobia, phonophobia, kinesophobia, nausea and throbbing pain) as is typically reported in IIH headache [1, 4, 6]. All patients were adult, female with IIH in occular remission and chronic moderate/severe headaches (≥15 per month) that ≥3 prior conventional oral preventative treatments had failed (inadequate efficacy with appropriate dosing and treatment duration; intolerable adverse effects; contraindications preventing use [8]) and then suffered recurrence of papilloedema whilst on erenumab. In this cohort treatments that failed included tricyclic antidepressants, topiramate, propranolol, pizotifen, duloxetine, dosulepin and candesartan. These chronic migraine-like headaches were elibigle for treatment with the CGRP monoclonal antibody erenumab (Aimovig®, Novartis) within the National Health Service (NHS) headache service at University Hospital Birmingham NHS Foundation Trust, UK as part of a free of charge scheme (Novartis).

The pathway for erenumab delivery entailed a full headache and neuro-ophthalmology assessment prior to erenumab initiation, including optical coherence tomography (OCT) imaging and dilated slit lamp fundus assessment, which confirmed complete resolution of papilloedema (Table 1). Patients were then commenced on erenumab 70 mg four-weekly subcutaneous injection after a 30-day pre-treatment period including headache diary evaluation. In all cases the headache response (improvement in monthly headache days and/ or severity) was greater than 30% but less than 50% and hence, as per the local pathway, the dose was increased to 140 mg at 3 months. Patients were seen 3-monthly over a 12-month period. Erenumab initiation (after assessment of a 30-day pre-treatment period) and follow-up visits involved headache diary evaluation of monthly moderate/severe headache days (MmsHD) (moderate to severe headaches lasting > 4 h), all monthly headache days (MHD) (headaches of any severity lasting > 30 min), monthly analgesic frequency, headache severity, disability (Headache Impact Test − 6, HIT-6) and IIH symptoms (Table 1). All patients had a stable course and substantial history of debilitating migrainous headaches prior to treatment initiation evident from the high burden of monthly headache days 12 months prior to treatment (Table 1).

**Table 1:** Patient characteristics at erenumab initiation

	P1	P2	P3	P4	P5	P6	P7	Mean (SD)
Age (years)	42	34	46	24	36	25	25	33.1 (8.2)
Duration of IIH (years)	2	2	1	1	4	2	5	2.4 (1.5)
Headache exacerbation at IIH diagnosis	+++	+++	+++	+++	+++	+++	+++	
Duration of headaches from resolution of papilloedema to initiation of erenumab (months)	12	3	3	3	24	6	3	7.7 (7.9)
Preventative drug class failure ^a^	4	3	3	4	5	4	3	3.7 (0.8)
Preventative drug class trial ^b^	3	2	1	0	4	1	1	1.7 (1.4)
Migraine family history	yes	no	missing	no	no	yes	no	
Migraine prior to IIH	yes	yes	yes	no	no	yes	no	
MmsHD 12 months prior	12	20	16	16	14	8	14	14.3 (3.5) *p* = 0.778
MmsHD at erenumab initiation	13	17	15	20	14	11	12	14.6 (3.1)
MHD 12 months prior	30	30	30	30	28	30	20	28.3 (3.7) *p* = 0.593
MHD at erenumab initiation	30	30	27	30	30	30	25	28.9 (2.0)
Headache severity (NRS) at erenumab initiation	5.7	5.2	7.2	7.3	5.2	4.5	7.1	6.0 (1.2)
Monthly analgesic days at erenumab initiation	2	6	6	20	7	4	12	8.1 (6.1)
HIT-6 score at erenumab initiation	68	65	70	75	66	68	63	67.9 (3.9)
Frisén grade at erenumab initiation (both eyes)	0	0	0	0	0	0	0	0
OCT global average RNFL thickness at Erenumab initiation worst eye (μm) ^c^	118	93	123	113	107	118	103	110.7 (10.4)
BMI at erenumab initiation	31	46.2	35.2	37.3	42.1	29.4	28.8	35.7 (6.6)
IIH characteristics at erenumab initiation	B, T	B, T	B, T	B, D, T	B, D, T	D, T	B, T	
Acetazolamide at erenumab initiation	no	no	no	no	no	500 mg	no	

Patient clinical characteristics at erenumab initiation. Monthly values refer to the 30-day pre-treatment period. *, *p* values indicate changes from the 30-day pre-treatment period prior to erenumab initiation

^a^Failure defined as any of: inadequate efficacy with appropriate dosing and treatment duration; intolerable adverse effects; contraindications preventing use

^b^Trial defined as any of: inadequate efficacy with appropriate dosing and treatment duration; intolerable adverse effects

^c^Heidelberg Engineering SPECTRALIS, Heidelberg, Germany

+++ severe

Abbreviations: *P* Patient, *IIH* Idiopathic intracranial hypertension, *MmsHD* Monthly moderate/severe headache days, *MHD* Monthly headache days, *NRS* Numeric Rating Scale (0 = no pain to 10 = worst imaginable pain), *HIT-6* Headache impact test-6, *BMI* Body mass index, *B* Blurred vision, *D* double vision, *T* Tinnitus, *V* Visual obscurations, *OCT* Optical coherence tomography, *RNFL* Retinal nerve fiber layer

### Case 1

A 42 year old woman first presented with severe increased headaches with visual obscurations, double vision, tinnitus and was noted to have papilloedema. The headaches had migraine-like characteristics. She had a past history of occasional episodic migraines, asthma and fibromyalgia and a family history of migraines. Her body mass index (BMI) was 33 kg/m^2^ and her LP opening pressure was 34 cm CSF with no alternative cause of raised ICP on brain imaging (MRI Head and MR Venogram). A diagnosis of IIH was made [6] and she was commenced on acetazolamide (later stopped due to side effects) and advised to lose weight. The papilloedema resolved but debilitating headaches continued over the subsequent 12 months. Therapeutic trials of topiramate, amitriptyline and propranolol failed (pizotifen was contraindicated) and she was then commenced on erenumab. Headaches substantially improved (Table 2) but she developed worsening of her visual disturbances similar to that experienced at presentation and on review was noted to have a papilloedema relapse (her BMI had also increased) (Table 2). An LP revealed an opening pressure of 31 cm CSF (followed by post-LP headache exacerbation for 1 week) and she was commenced on acetazolamide and advised to lose weight with subsequent improvement of papilloedema. Her headaches remained controlled throughout the following year despite the relapse of IIH (Table 2).

**Table 2:** Patient clinical outcomes

	P1	P2	P3	P4	P5	P6	P7	Mean (SD)
MmsHD change at 3 months	− 3	− 6	− 9	− 9	− 2	− 6	− 4	− 5.6 (2.8) *p* = 0.002
MmsHD change at relapse	− 5	− 13	− 6	− 13	− 7	− 10	− 6	−8.6 (3.4) *p* < 0.001
MHD change at 3 months	− 20	0	− 18	− 12	− 15	−16	− 4	−12.1 (7.4) *p* = 0.028
MHD change at relapse	− 22	0	0	−15	− 10	− 24	− 5	− 10.9 (9.9) *p* = 0.043
Headache severity (NRS) change at 3 months	2	−0.9	0.2	−2.6	0.1	0.1	−1.7	−0.4 (1.5) *p* = 0.497
Headache severity (NRS) change at relapse	2.7	−1.4	−0.8	−2.7	− 1.8	− 0.4	−2.4	− 1.0 (1.8) *p* = 0.253
Monthly analgesic days change at 3 months	3	−1	− 6	− 18	0	− 4	−9	− 5.0 (7.0) *p* = 0.107
Monthly analgesic days change at relapse	0	0	−5	−18	−7	1	−10	−5.6 (6.9) *p* = 0.075
HIT-6 score change at 3 months	− 5	−11	− 4	− 13	1	− 8	2	− 5.4 (5.7) *p* = 0.045
HIT-6 score change at relapse	− 5	− 13	−4	−3	0	−18	− 2	−6.4 (6.6) *p* = 0.041
Frisén grade at relapse (worst eye)	4	2	3	1	1	1	1	1.9 (1.2)
OCT global average RNFL thickness at relapse worst eye (μm) ^a^	326 ^b^	140 ^b^	174 ^b^	132 ^c^	116 ^c^	151 ^c^	202 ^c^	177.3 (71.4) *p* = 0.043
BMI at relapse	31.3	48.5	38.7	38	44.6	36.7	30.5	38.3 (6.5) *p* = 0.025
BMI change	0.3	2.3	3.5	0.7	2.5	7.3	1.7	2.6 (2.3)
IIH characteristics at relapse	B, T, V	B, T	B, T, V	B, D, T	B, D, T	B, D, T	B, T	
Side effects		CO		HT, I	I	CO, MC		

Clinical outcomes and changes compared to erenumab initiation and the 30-day pre-treatment period *, *p* values indicate changes from erenumab initiation and the 30-day pre-treatment period

^a^Heidelberg Engineering SPECTRALIS, Heidelberg, Germany

^b^At 6 months since commencing erenumab

^c^At 12 months since commencing erenumab

Abbreviations: *P* Patient, *IIH* Idiopathic intracranial hypertension, *MmsHD* Monthly moderate/severe days, *MHD* Monthly headache days, *NRS* Numeric Rating Scale (0 = no pain to 10 = worst imaginable pain), *HIT-6* Headache impact test-6, *BMI* Body mass index, *B* Blurred vision, *D* Double vision, *T* Tinnitus, *V* Visual obscurations, *OCT* Optical coherence tomography, *RNFL* Retinal nerve fiber layer, *CO* Constipation, *HT* Hair thinning, *I* Itch, *MC* Muscle cramps

### Case 2

**Fig. 1:**
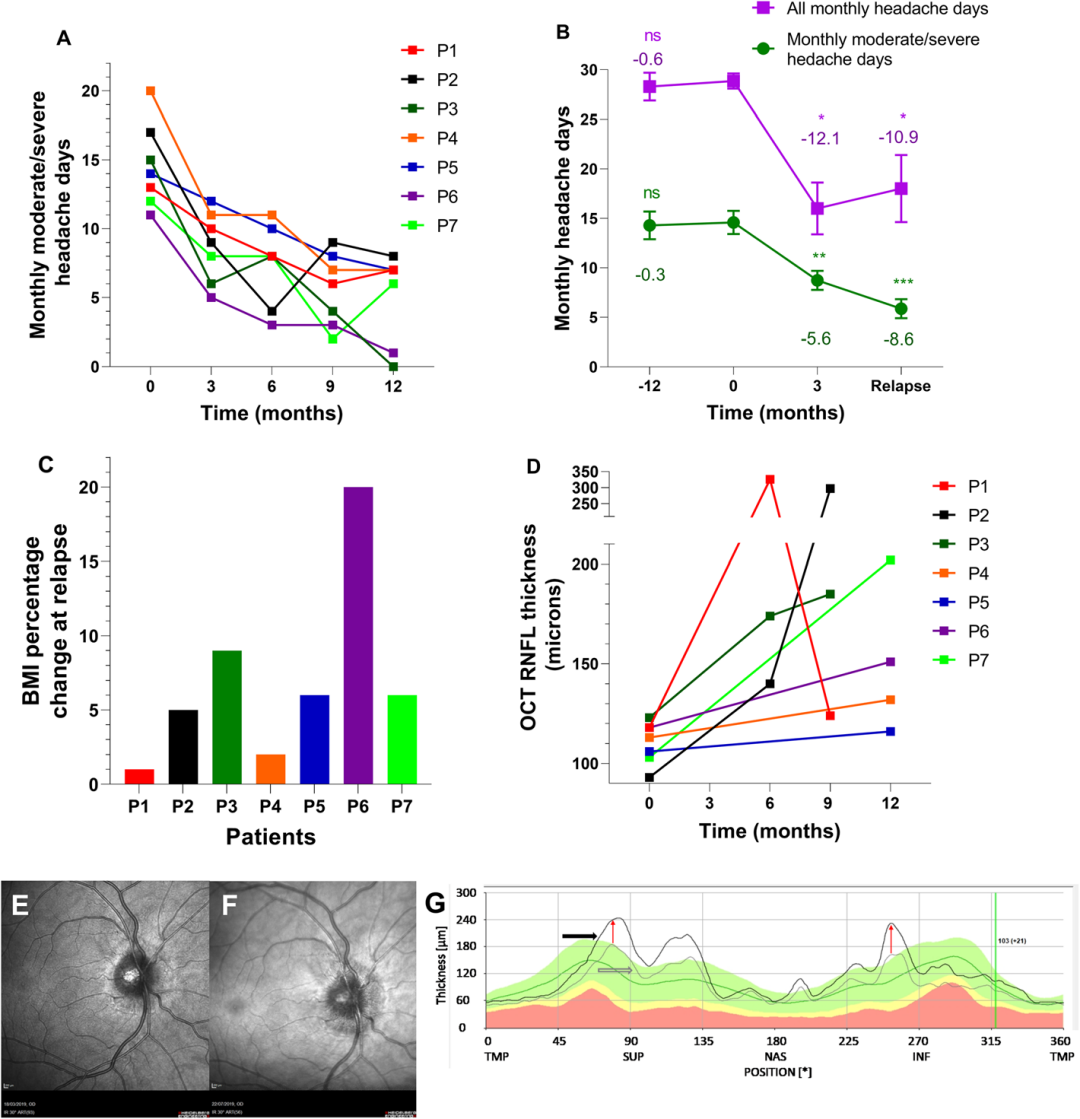
Headache days, BMI and optical coherence testing of patients. **a** Monthly moderate/severe headache days (MmsHD) at clinical assessment time-points. Each patient is represented by different colour. Relapse point is represented by empty circle or triangle large point for each patient. **b** Mean number of monthly moderate/severe headache days (MmsHD) and total monthly headache days (MHD) at − 12 months, erenumab initiation (0 months), 3 months and at relapse. Error bars represent standard error of the mean (SEM). T-test performed for changes compared to erenumab initiation for MmsHD and Wilcoxon signed ranks test performed for changes compared to erenumab initiation for MHD. ***P < 0.001 compared to erenumab initiation, ** *P* < 0.01 compared to Erenumab initiation, * *P* < 0.05 compared to Erenumab initiation. **c** Body mass index percentage change at time of relapse compared to erenumab initiation (substantial fluctuations in weight were possible in between formal clinical assessments, but were not measured). Each patient is represented by different colour. **d** Optical coherence tomography (OCT) global average peripapillary retinal nerve fibre layer (pRNFL) thickness at clinical assessment time-points (0 months represents erenumab initiation time-point). Each patient is represented by different colour. **e** Infrared image of the right eye at erenumab initiation (Heidelberg Engineering SPECTRALIS, Heidelberg, Germany) for Patient 2 (P2). This shows no papilloedema. **f** Infrared image of the right eye at 6 months Patient 2 (P2). This shows recurrence of papilloedema. **g** Graph of OCT cross-sectional pRNFL thickness derived from 12° ring scan centred on the optic disc (Heidelberg Engineering SPECTRALIS, Heidelberg, Germany) for Patient 2 (P2). Black line shows the cross-sectional pRNFL thickness of the six-month scan (relapse), with the grey line showing the same information for the erenumab initiation scan. The difference between these lines (red arrows) indicates the magnitude of increase in pRNFL thickness between these scans, demonstrating relapse of IIH and recurrence of active papilloedema. The shaded green area indicates the proprietary ‘normal’ range for pRNFL thickness. Abbreviations: MmsHD, Monthly moderate/severe headache days; MHD, Monthly headache days; OCT, Optical coherence testing; RNFL, retinal nerve fibre layer; TMP, Temporal; SUP, Superior; NAS, Nasal; INF, Inferior

A 34 year old woman initially presented with increased debilitating headaches, blurred vision, visual obscurations, tinnitus and was noted to have papilloedema by the optometrist. The headaches had migraine-like characteristics. She had a past history of episodic migraine but no family history of migraines. Her BMI was at 49 kg/m^2^ (reported weight gain of 30 kg over the preceding 5 years). After normal neuro-imaging, CT Head and CT Venogram (apart from radiological signs of raised ICP), she had an LP opening pressure of 34 cm CSF. A diagnosis of IIH was confirmed [6] and she was commenced on acetazolamide and advised to lose weight. She developed side effects from acetazolamide, this was discontinued. Therapeutic trials of topiramate and amitriptyline failed (beta blockers and pizotifen were contraindicated) and her chronic headaches remained. Following weight loss her papilloedema resolved but migraine-like headaches persisted with similar frequency and severity for over 12 months and she was subsequently commenced on erenumab. Headaches then substantially improved (Table 2). During follow-up she reported weight gain and symptoms of visual disturbances. She was noted to have a papilloedema relapse (Fig. 1). She was offered acetazolamide but declined and a referral to a community weight management program was made. Following further weight gain, after gall bladder surgery, her papilloedema further worsened and she underwent ventriculo-peritoneal shunt surgery to preserve vision. Her headaches since erenumab initiation remained improved despite the IIH relapse and shunt surgery.

### Case 3

A 46 year old woman presented with substantially increased headache symptoms, with migraine-like characteristics, and was noted to have papilloedema. She had a past history of episodic migraine for 20 years, depression but no known family history of migraine. Her BMI was 39 kg/m^2^ and no alternative cause of raised ICP on brain imaging (MRI Head and MR Venogram). LP recorded an opening pressure of 36cmCSF and a diagnosis of IIH was made [6]. She was offered acetazolamide and advised to lose weight. Following weight loss, papilloedema resolved but her chronic migraine-like headaches remained consistently severe for at least 12 months (Table 2). Headaches did not improve with a therapeutic trial of amitriptyline. Other preventative therapies were contraindicated (topiramate and beta-blockers due to severe depression and pizotifen due to the risk of weight gain) and erenumab was commenced. Headaches substantially improved (Table 2), but she developed visual disturbances (obscurations) and was noted to have recurrent active papilloedema. She was commenced on acetazolamide and advised to lose weight. Headaches remained improved (Table 2) despite return of papilloedema.

### Case 4

A 24 year old woman first presented with worsening headaches and visual disturbances and was noted to have papilloedema with a BMI of 38 kg/m^2^. The headaches had a migraine-like phenotype. She had a history of occasional tension type headaches, bipolar disorder and no family history of migraines. With no alternative cause of raised ICP on brain imaging (MRI Head and MR Venogram) an LP was performed revealing an opening pressure of 40 cm CSF and a diagnosis of IIH was made [6]. She had further LPs with similar opening pressures in the short term with no sustained headache improvement and was commenced on acetazolamide. At subsequent review her papilloedema was in remission and she had already stopped her acetazolamide due to side effects. She was, however, still suffering with ongoing disabling chronic migraine-like headaches for over 12 months since IIH diagnosis. Due to risk of deteriorating mood and weight gain topiramate and tricyclics/beta-blockers were contraindicated, respectively. Candesartan was also contraindicated due to postural vasovagal episodes and she was commenced on erenumab. Her headaches substantially improved (Table 2). Subsequently, visual obscurations returned and she was noted to have a relapse of her papilloedema whilst still maintaining the improvement in her headaches. She was offered acetazolamide, advised to lose weight and her papilloedema did not worsen. Her headaches remained in remission during the IIH recurrence.

### Case 5

A 36 year old woman, with a BMI of 41 kg/m^2^, initially presented with debilitating headaches, tinnitus and visual disturbances. The optometrist noted papilloedema. Headaches had a migraine-like phenotype. She had a past medical history of low mood, familial structural renal tract abnormalities and no family history or prior history of migraines. Following normal neuro-imaging, CT Head and CT venogram (apart from radiological signs of raised ICP), an LP was performed with an opening pressure recorded at 37 cm CSF. A diagnosis of IIH was given [6] and due to the risk of renal impairment acetazolamide was not recommended and she was referred to a community weight management program which successfully helped her reduce her BMI to 30 kg/m^2^ with concurrent resolution of her papilloedema. Her chronic migraine-like headaches persisted over 12 months despite therapeutic trials of amitriptyline, topiramate, dosulepin and candesartan with inadequate efficacy or intolerable side effects. She was commenced on erenumab with a marked improvement in headaches (Table 2). Unfortunately her weight increased (to a BMI 42 kg/m^2^) and her papilloedema recurred. However, her headaches did not increase as they previously have when she initially presented with IIH (Table 2). She was again referred to a community weight management program which was successful in helping her reduce her weight and her papilloedema improved.

### Case 6

A 25 year old woman originally presented with marked worsening of headaches and tinnitus, papilloedema was noted. Her BMI was 35 kg/m^2^. The headache phenotype was migraine-like. She had a past history of episodic migraines, hypermobility, back pain, Raynaud’s syndrome, depression, low vitamin D and family history of migraines. No alternative cause of raised ICP was found on brain imaging (MRI Head and MR Venogram) and an LP was performed with an opening pressure of 35 cm CSF and the diagnosis of IIH was made [6]. She was given advice on weight loss and commenced on acetazolamide. Following successful weight loss her papilloedema resolved despite persistence of her chronic migraine-like headaches over 12 months. Due to resolution of papilloedema and associated side effects, acetazolamide was stopped. She had tried duloxetine for low mood with no effect on her persistent headaches. Due to risk of deteriorating mood and weight gain topiramate and tricyclics/beta-blockers were contraindicated, respectively and was commenced on erenumab. Headaches improved on erenumab (Table 2) but she then developed increasing blurred vision in the context of weight gain. On examination a relapse of papilloedema was noted. Acetazolamide was commenced and weight loss advice was provided. The headaches remained controlled throughout the IIH relapse.

### Case 7

A 25 year old woman (BMI of 31 kg/m^2^) presented with disabling increasing headaches and papilloedema was noted. The headaches had a migraine-like phenotype. She did not have any significant past medical history and no family history of migraines. Neuro-imaging (MRI Head and MR Venogram) did not reveal an alternative cause of raised ICP and an LP recorded an opening pressure of 54 cm CSF (followed by post-LP headache exacerbation for 1 week). A diagnosis of IIH was made [6]. She was commenced on acetazolamide and advised to lose weight. Neither acetazolamide nor topiramate were tolerated and did not improve her chronic migraine-like headaches that remained over 12 months. Following weight loss, the papilloedema resolved but migraine-like headaches persisted. Due to risk of weight gain, tricyclics and beta-blockers were contraindicated and erenumab was initiated which improved her headaches (Table 2). At follow-up weight gain was noted and clinical examination revealed return of papilloedema. She was commenced on acetazolamide and advised to lose weight. Papilloedema subsequently improved. Her headaches remained controlled on erenumab despite the IIH relapse.

### Summary results

We have reported seven patients with IIH who had disabling long term headaches with migraine-like characteristics (mean (SD) IIH duration 2.4 (1.4) years) persisting despite complete resolution of papilloedema. Monthly headache burden was significantly reduced following initiation of a CGRP monoclonal antibody therapy (Table 2). Of principle interest here is the lack of headache symptoms when raised ICP retuned, as evidenced by recurrent papilloedema, whist they were taking the CGRP monoclonal antibody, despite headache being the dominant feature at initial IIH presentation and indicating exacerbations of IIH in the past. The relapse of IIH was identified at follow up due to patients’ describing minimal or subjective changes in IIH symptoms (blurred vision, double vision, transient visual obscurations, tinnitus) or weight gain (Table 1) which prompted slit-lamp examination and OCT imaging, which confirmed the recurrence of papilloedema (Table 2, Fig. 1). Improved headache burden was also noted at subsequent time points despite ongoing papilloedema (Fig. 1). Erenumab reduced the mean frequency of MmsHD by 38% at 3 months, 49% at 6 months, 63% at 9 months and 65% at 12 months compared to the 30-day pre-treatment period. There was a reduction in mean frequency in overall MHDs by 45% at 3 months, 30% at 6 months, 50% at 9 months and 42% at 12 months. There were no serious side effects of treatment reported at the 3-monthly assessments (over 12 months) and none of the patients withdrew from erenumab.

## Discussion

Erenumab significantly reduced the mean frequency of MmsHD at the 3-monthly time-points up to 12 months compared to the 30-day pre-treatment period, with corresponding significant reductions in the total MHD and HIT-6 score, despite the recurrence of active IIH and relapse of papilloedema (Table 2). Whilst spontaneous fluctuation in headaches are well described, we suggest that the improvements noted in these subjects are unlikely to represent a fluctuating disease course as the headaches had been stable and severe (for at least 12 months) prior to initiation of Erenumab and then showed sustained improvement (over the forthcoming 12 months) after starting erenumab.

The mechanisms driving headache in patients with IIH have not been elucidated and treatment approaches have no evidence basis [1, 4, 5]. Headaches in IIH are typically chronic migraine-like, drive disability, and are a patient prioritised key area for mechanistic research and treatment [3–5]. CGRP is a key modulator of migraine headaches [9] and this case series suggests evidence for the role of CGRP as an important nociceptive stimulus in IIH headaches. Four out of seven patients had a pre-existing migraine diagnosis. However all patients presented with a severe headache exacerbation at the initial presentation of IIH, indicating that their headaches exacerbation were temporarily linked with raised ICP and fulfilled the International Classification of Headache Disorders diagnostic criteria 3b for IIH headache [10]. Following ocular remission, headaches persisted (as frequently occurs in IIH) [11] and were successfully treated as chronic migraine-like headaches with erenumab (after ≥3 prior conventional oral preventative treatment failures). In these patients, in whom headache has been the presenting symptom indicating elevated ICP at diagnosis, erenumab effectively controlled headaches even when IIH relapsed with return of papilloedema. In the 4 patients with pre-existing migraine we can’t exclude that in these subjects’ headaches were driven by co-morbid migraine.

The therapeutic reduction of headache was noted in IIH patients in ocular remission, but more importantly also during the subsequent phase of recurrent active IIH, suggesting that headaches driven by raised ICP may involve CGRP release.

Exacerbation of headache is a common trigger for IIH patients to seek medical review, typically indicated recurrence of IIH and papilloedema. However, development of active papilloedema in this series of IIH patients treated with a CGRP monoclonal antibody was not associated with exacerbation of headache. Whilst the enduring control of headaches, even during a period of actively elevated ICP with papilloedema, is symptomatically advantageous to the patients there is also an important caution. In these cases, the headaches were controlled and did not recur to warn of an IIH disease relapse hence there could be a risk that relapse of IIH could be missed. Funduscopic review is typically reduced in frequency in IIH patients in whom IIH has resolved (typical follow up intervals recommended in the IIH guidelines) [1]. But in IIH patients who respond to CGRP monoclonal antibody therapy for headache, we would recommend ongoing regular fundoscopy, with a low threshold for a complete neuro-ophthalmic assessment and pharmacovigilance monitoring as recurrence of headache may not occur to warn of an IIH relapse. In these cases, mild subjective changes in IIH symptoms and weight gain were markers of IIH relapse (in the absence of headache recurrence). It is important not to miss recurrence of papilloedema, as patients require visual function monitoring and management to mitigate the risk of visual loss [6]. In the setting of known IIH we feel it is unlikely that the papilloedema was attributed to the erenumab treatment itself, but this cannot be excluded.

These cases may provide the first insight that CGRP may be a key mechanistic driver for IIH headaches when raised ICP is present, manifesting as papilloedema. Headache therapies are a patient priority and an unmet need in IIH with no previous trials of headache preventative therapies in IIH [5]. CGRP may represent a therapeutic target for raised ICP IIH headaches and clinical trials investigating CGRP-specific agents would be of interest.

## Clinical implications

- CGRP monoclonal antibodies successfully treat resistant chronic migraine-like headaches in those with IIH in whom their papilloedema had resolved.
- CGRP may have a mechanistic role in driving raised ICP headaches in active IIH.
- The therapeutic potential of CGRP monoclonal antibodies for IIH headache warrants further evaluation in a clinical trial.

## Data Availability

The data that support the findings of this case series are available from the corresponding author on reasonable request.

## Abbreviations

IIH: Idiopathic Intracranial Hypertension
ICP: Raised intracranial pressure
CGRP: Calcitonin gene related peptide
NHS: National Health Service
OCT: Optical coherence tomography
MmsHD: Monthly moderate/severe headache days
MHD: Monthly headache days
HIT-6: Headache Impact Test − 6
